# Effects of polystyrene nano- and microplastics on human breast epithelial cells and human breast cancer cells

**DOI:** 10.1016/j.heliyon.2024.e38686

**Published:** 2024-10-04

**Authors:** Maximilian Schnee, Mareike Sieler, Jessica Dörnen, Thomas Dittmar

**Affiliations:** a Institute of Immunology, Center for Biomedical Research and Education (ZBAF), Witten/Herdecke University, Witten, Germany; b Faculty of Medicine, Ruhr University Bochum, Bochum, Germany

**Keywords:** Polystyrene nano- and microplastics, Human breast epithelial cells, Human breast cancer cells, Cell migration, Cancer stem cell properties, Cell fusion

## Abstract

The continuous littering of the environment with plastic and the resulting nano- and microplastics produced from various processes are ever-increasing problems. These materials also affect humans, as the absorption and accumulation of nano- and microplastics and their effects on health have thus far been rarely researched, which also applies to cancer. In the present study, the absorption of different sizes of polystyrene (PS) nano- and microplastics (PS particles) into human breast epithelial cells and human breast cancer cells was investigated. Subsequently, how the proliferation, colony and mammosphere formation abilities, cell fusion and migration of the cells were influenced by the PS particles were investigated. Our data revealed granularity-, dose- and cell line-dependent absorption of the PS particles, with the highest absorption observed in the MDA-MB-231-DSP1-7 cells and the lowest in the M13SV1_Syn1-DSP8-11 cells. Neither the colony-forming ability nor the cell fusion activity increased with the addition of PS particles. In contrast, slight, partially significant stimulatory effects on both proliferation and cell migration were observed, although these effects depended on the particle quantity and size and the cell line used. In summary, PS particles are absorbed by human breast epithelial and human breast cancer cells and influence cells that may be associated with cancer progression.

## Highlights

• PS particles are internalized in a dose-, granularity- and cell line-dependent manner.
• The proliferation of human breast cancer cell lines is influenced by PS particles.
• The formation of human breast cancer cell mammospheres is triggered by PS particles.
• PS particles promote breast epithelial cell and breast cancer cell migration.

## 1 Introduction

Nano- and microplastic pollution is one of the major challenges of the current age, affecting the climate, environment and human health [1]. One key reason for the increase in pollution is the general rise in demand for plastic products [2]. Most of these products are single-use plastic products, such as cutlery, plates, bottles and packaging, which produce vast amounts of plastic waste. The finding that only a portion of the plastic waste produced is properly disposed of and recycled in incinerators or landfills, meaning that a considerable amount still ends up in the environment, is extremely problematic [3]. Plastics are very durable and weather resistant. These properties are both advantages and disadvantages, as plastic does not simply disintegrate into its constituent parts. In nature, larger plastic particles are broken down by thermal, mechanical and other physical processes (UV light, waves, and wind) or by biodegradation (e.g., decomposition by viruses, bacteria, and algae) and ultimately become nano- and microplastics [4,5]. Nano- and microplastics in particular pose major problems for humans and nature, as they are easily dispersed due to their size and occur ubiquitously in various environmental media, such as water, air and soil [6].

Nanoplastics are defined by a particle diameter of less than 1 μm [7], whereas microplastics are defined by a particle diameter of less than 5 mm [8]. Primary nano- and microplastics are manufactured for a variety of applications, such as microfibers for clothing, abrasive beads for toothpastes, and glitter for cosmetics [[9], [10], [11]]. In contrast, secondary nano- and microplastics arise mainly from the degradation of larger plastic fragments through natural weathering processes, including tire wear, plastic bags, plastic bottles and fishing nets [6,12]. While the negative effects of microplastics on various ecosystems are now well documented, the results from correlative studies in people, model animals and cultured cells exposed to high concentrations of nano- and microplastics suggest that the effects of nano- and microplastics could include provoking immune and stress responses, inducing reproductive and developmental toxicity and potentially playing a role in cancer [4,[13], [14], [15], [16], [17]].

Initially, only very small plastic particles (diameter <0.1 μm) were assumed to be absorbed in the gastrointestinal tract (oral) and the respiratory tract (inhalation), whereas larger microplastic particles (>90 %) would be excreted via feces or mucus [18,19]. However, nano- and microplastic particles of 0.7–10 μm in size have been detected in the blood, placenta, cells and even in human breastmilk, indicating that these particles can pass through the respiratory and gastrointestinal barriers and accumulate in the body, despite their larger diameter [13,[20], [21], [22], [23]]. For example, the accumulation of fluorescent 5 μm and 20 μm polystyrene (PS) microplastics in the liver, kidney and gut was observed in murine models and was further associated with microplastic particle size-dependent tissue accumulation kinetics and distribution patterns [23]. Moreover, animal studies by Liang et al. indicated that PS micro- and nanoparticles jointly induced intestinal barrier dysfunction via reactive oxygen species (ROS)-mediated epithelial cell apoptosis concomitant with their dynamic biodistribution in the blood and various organs, such as the spleen, kidney, heart, brain, lungs and liver [24]. This finding might explain how larger nano- and microplastics could pass through the intestinal barrier. The daily absorption of nano- and microplastics can only be approximated, as it depends on the extent to which water, foodstuffs and cosmetics are contaminated with them. According to estimates, the daily absorption of nano- and microplastics is approximately 5 ml, which corresponds to a particle count of 4594–94,500 particles [25,26]. Even if a large proportion of these plastic particles were to be excreted again, the remainder would accumulate in the body over a longer period of time.

The absorption of nano- and microplastics derived from different polymers in a particle size- and dose-dependent manner has been demonstrated for several normal cell types and cancer cells, such as human mast cells (HMC-1), human dermal fibroblasts (HDFs), peripheral blood mononuclear cells, intestinal epithelial cells, CCD-18Co human colorectal fibroblasts, MCF7 and MDA-MB-231 human breast cancer cells, A549 human lung cancer cells, and HT-29 and Caco2 human colorectal adenocarcinoma cells [3,15,16,[27], [28], [29], [30]]. Interestingly, surface-functionalized PS nanoparticles were observed inside A549 lung carcinoma cells, which correlated with decreased cell viability and enhanced genotoxicity due to micronuclei formation and ROS production [16]. These findings indicate that nano- and microplastic particles may increase the genomic instability of tumor cells, which is a hallmark of solid cancers [31]. Similarly, irregularly shaped and sharp-edged polypropylene microparticles, which form naturally in the environment, increase the expression of metastasis-related genes and cytokines in breast cancer cells [15]. These data suggest the putative involvement of nano- and microplastics in cancer metastasis, which is another hallmark of solid tumors [31].

In the present study, we investigated the effects of PS nano- and microparticles (PS particles) on a human breast epithelial cell line and two human breast cancer cell lines. PS is thermoplastic and is used to produce toothbrushes, food containers, disposable plastic cutlery and dinnerware, or Styrofoam [32,33]. It is found in placental tissue and in breastmilk [20,22], which indicates that it can accumulate in breast tissue and should also accumulate in breast cancer tissues. Due to incomplete polymerization, PS can harbor styrene dimers and styrene trimers, which are suspected to have estrogenic activity [34] and thus may affect normal breast tissue or estrogen receptor-positive breast cancer. However, both in vitro and in vivo testing revealed no estrogenic activity of styrene oligomers [34]. Data from Park and colleagues revealed that polypropylene (PP) microplastics likely promote metastatic features in human MDA-MB-231 and MCF-7 breast cancer cells by increasing metastasis-related gene expression [15]. The same cell lines were used in the work of Voronovic and colleagues, who studied the absorption of rare-earth-doped nanoparticles, which are promising candidates for bioimaging, therapy, and drug delivery [29]. However, PS differs from PP and rare-earth-doped nanoparticles; thus, the effects of PS particles on human breast epithelial tissue and breast cancer tissue remains unclear.

In the present work, the absorption of PS particles with different granularities and concentrations by human M13SV1_Syn1-DSP8-11 breast epithelial cells and HS578T-DSP1-7 and MDA-MB-231-DSP1-7 human breast cancer cells was analyzed. Similarly, the effects of the absorbed PS particles on prospective (cancer) stem cell properties, migratory behavior and the ability to fuse were further investigated. HS578T and MDA-MB-231 breast cancer cells are triple-negative breast cancer (TNBC) cell lines [35]. TNBC represents an aggressive subtype of breast cancer that is associated with a poor prognosis for affected patients [36,37]. This subtype lacks estrogen receptor, progesterone receptor and HER2 expression; thus, appropriate targets for therapy are lacking. Thus, we were interested in whether certain tumor-promoting capacities of TNBC cells were positively triggered by PS particles.

## 2 Materials and methods

### 2.1 Cell culture

M13SV1_Syn1-DSP8-11 human breast epithelial cells and HS578T_DSP1-7 and MDA-MB-231_DSP1-7 human breast cancer cells were generated and cultivated as described previously [38]. Briefly, M13SV1_Syn1-DSP8-11 cells were cultured in RPMI 1640 media (PAN Biotech GmbH, Aidenbach, Germany) supplemented with 10 % fetal calf serum (FCS; PAN Biotech GmbH, Aidenbach, Germany), 100 U/ml penicillin/0.1 mg/ml streptomycin (PAN Biotech GmbH, Aidenbach, Germany), 10 μg/ml recombinant human epidermal growth factor (rhEGF), 5 μg/ml human recombinant insulin, 0.5 μg/ml hydrocortisone, 4 μg/ml human transferrin, 10 nM β-estrogen, 2 μg/ml puromycin and 200 μg/ml hygromycin-B (rhEGF, insulin, hydrocortisone, transferrin, β-estrogen and puromycin were purchased from Merck KGaA, Darmstadt, Germany, whereas hygromycin-B was purchased from PAN Biotech, Aidenbach, Germany). HS578T-DSP1-7 human breast cancer cells were maintained in RPMI 1640 media (PAN Biotech GmbH, Aidenbach, Germany) supplemented with 10 % fetal calf serum (FCS; PAN Biotech GmbH, Aidenbach, Germany), 100 U/ml penicillin/0.1 mg/ml streptomycin (PAN Biotech GmbH, Aidenbach, Germany) and 200 μg/ml hygromycin-B (PAN Biotech GmbH, Aidenbach, Germany). MDA-MB-231-DSP1-7 human breast cancer cells were cultured in DMEM (PAN Biotech GmbH, Aidenbach, Germany) supplemented with 10 % fetal calf serum (FCS; PAN Biotech GmbH, Aidenbach, Germany), 100 U/ml penicillin/0.1 mg/ml streptomycin (PAN Biotech GmbH, Aidenbach, Germany) and 200 μg/ml hygromycin-B (PAN Biotech GmbH, Aidenbach, Germany). All the cells were cultured in a humidified atmosphere with 5 % CO_2_ at 37 °C. A detailed description of the vector design and generation is provided in Ref. [38]. The cell lines were authenticated via PCS single-locus technology (Eurofins Genomics, Ebersberg, Germany). The obtained STR profiles of HS578T-DSP-17 and MDA-MB-231-DSP1-7 breast cancer cells are consistent with the STR profiles of both cell lines in the Cellosaurus database (HS578T-DSP1-7: CVCL_0332; MDA-MB-231-DSP1-7: CVCL_0062; www.cellosaurus.org). As parental M13SV1 breast epithelial cells were derived from primary mammary tissue [39], no STR data were available from public databases. All the cell lines used in this study were routinely tested for possible mycoplasma contamination using a Mycoplasma PCR Detection Kit (BioCat GmbH, Heidelberg, Germany) and were mycoplasma-negative.

### 2.2 Microplastic particle sizes and concentrations

Three different sizes of monodisperse green fluorescent-labeled PS particles (0.5 μm, 1.0 μm and 4.5 μm) were used in this study. The 0.5 and 4.5 μm PS particles were purchased from microParticles GmbH, Berlin, Germany, whereas the 1.0 μm PS particles were ordered from Merck KGaA, Darmstadt, Germany. The stock solution of the 0.5 μm PS particles contained 1.21 × 10^11^ p/ml, that of the 1 μm PS particles contained 4.74 × 10^10^ p/ml, and that of the 4.5 μm PS particles contained 2.02 × 10^8^ p/ml. Five different particle concentrations were prepared by dilution in phosphate-buffered saline (PBS) to obtain concentrations of 64,000 p/ml, 32,000 p/ml, 16,000 p/ml, 8,000 p/ml and 4,000 p/ml for experiments.

### 2.3 Cell‒cell fusion assay

The dual split (DSP) assay [40,41] was applied for the visualization and quantification of cell‒cell fusion events between human M13SV1_Syn1-PuroR-DSP8-11 breast epithelial cells and human HS578T-DSP1-7 and MDA-MB-231-DSP1-7 breast cancer cells in accordance with a previous study [38]. Each cell line expresses one half of a green fluorescent protein (GFP) (either DSP1-7 or DSP8-11). When cells expressing DSP1-7 and DSP8-11 fuse with each other, the two halves of the GFP reunite and form a functional GFP that can be detected by fluorescence microscopy and flow cytometry. M13SV1_Syn1-DSP8-11 breast epithelial cells (1 × 10^4^) were either cocultured with 3 × 10^4^ HS578T-DSP1-7 or 3 × 10^4^ MDA-MB-231-DSP1-7 breast cancer cells in duplicate wells of a 96-well plate (Sarstedt, Nümbrecht, Germany) for 48 h in a humidified atmosphere at 37 °C with 5 % CO_2_. The medium (250 μl) was supplemented with the appropriate concentration of microparticles (64,000 p/ml and 4,000 p/ml for all three PS particle granularities). Untreated cells served as a control. Cell‒cell fusion was visualized using the IncuCyte® SX5 LiveCell Imaging system (Sartorius AG, Göttingen, Germany). Transmission light microscopy and green fluorescence images were captured every 4 h. This approach was only used for the qualitative analysis and visualization of cell‒cell fusion events and, thus, the data were not further analyzed.

### 2.4 Flow cytometry

The absorption of green fluorescent-labeled PS particles was quantified by flow cytometry using a FACSCalibur flow cytometer (BD Dickenson, Heidelberg, Germany). Briefly, 1 × 10^5^ cells (M13SV1_Syn1-DSP8-11, HS578T-DSP1-7 and MDA-MB-231-DSP1-7) were seeded in 6-well plates (Sarstedt, Nümbrecht, Germany) and cultivated in the appropriate medium (1.5 ml per well) for 24 h at 37 °C with 5 % CO_2_ in a humidified atmosphere. The medium was subsequently replaced with medium containing the appropriate concentration of microparticles (64,000 p/ml, 32,000 p/ml, 16,000 p/ml, 8,000 p/ml and 4,000 p/ml for all three PS particle granularities). Untreated cells served as controls. The cells were cultivated for an additional 24 h. Thereafter, the cells were harvested, washed twice with PBS and resuspended in 500 μl of PBS.

Flow cytometry was also used to quantify cell‒cell fusion (see *Cell‒cell fusion assay)*. The cocultured cells were harvested, washed twice with PBS and then resuspended in 250 μl of PBS. The number of green fluorescent cells was quantified by flow cytometry (FACSCalibur; Becton Dickenson, Heidelberg, Germany). In addition to untreated cells, freshly mixed M13SV1_Syn1-DSP8-11 + HS578T-DSP1-7 cells or M13SV1_Syn1-DSP8-11 + MDA-MB-231-DSP1-7 cells served as additional controls. WinMDI 2.9 software (http://www.cyto.purdue.edu/flowcyt/software/Winmdi.htm) was used to analyze the flow cytometry data.

### 2.5 Cell proliferation studies

Cell proliferation studies were performed using the IncuCyte® SX5 LiveCell Imaging system (Sartorius AG, Göttingen, Germany). M13SV1_Syn1-DSP8-11, HS578T-DSP1-7 and MDA-MB-231-DSP1-7 cells (2,500 cells per well) were seeded in triplicate in a 96-well plate (Sarstedt, Nümbrecht, Germany) and cultured in the appropriate medium (250 μl per well) for 24 h at 37 °C with 5 % CO_2_ in a humidified atmosphere. The medium was subsequently replaced with medium containing the appropriate concentration of microparticles (64,000 p/ml, 32,000 p/ml, 16,000 p/ml, 8,000 p/ml and 4,000 p/ml for all three PS particle granularities). Untreated cells served as controls. The cells were cultured for up to 72 h, and transmission microscopy images and green fluorescence images were captured every 4 h. IncuCyte® 21A software (Sartorius AG, Göttingen, Germany) was used to analyze the cell proliferation data.

### 2.6 Determination of the relative cell size

The relative sizes of the cell lines used (M13SV1_Syn1-DSP8-11, HS578T-DSP1-7 and MDA-MB-231-DSP1-7) were determined from Incucyte SX5 cell proliferation data. Therefore, a cell analysis mask was created in which individual cells were counted as objects. Incucyte® 21A software (Sartorius AG, Göttingen, Germany) was subsequently used to determine the average area of the counted objects, which was equal to the relative cell size.

### 2.7 Colony formation assay

M13SV1_Syn1-DSP8-11, HS578T-DSP1-7 and MDA-MB-231-DSP1-7 cells (200 cells per well) were seeded in 6-well plates (Sarstedt, Nümbrecht, Germany) and cultivated in the appropriate medium (1.5 ml per well) for 24 h at 37 °C with 5 % CO_2_ in a humidified atmosphere. Then, the medium was replaced with medium containing the appropriate concentration of PS particles (64,000 p/ml, 32,000 p/ml, 16,000 p/ml, 8,000 p/ml and 4,000 p/ml for all three PS particle granularities). Untreated cells served as controls. The cells were cultured for 12 days. Then, the medium was removed, and the cells were fixed with a 4 % paraformaldehyde solution (Merck KGaA, Darmstadt, Germany) for 10 min at room temperature. Thereafter, the fixed cells were stained with a 0.5 % crystal violet solution (Merck KGaA, Darmstadt, Germany) for 1 h. Finally, the fixed and stained cells were washed with H_2_O and air-dried. The 6-well plates were scanned, and the relative colony formation capacity was determined using Fiji (ImageJ) software (https://imagej.net/software/fiji/downloads).

### 2.8 Mammosphere formation assay

M13SV1_Syn1-DSP8-11, HS578T-DSP1-7 and MDA-MB-231-DSP1-7 cells (500 cells per well) were cultured in ultralow attachment 96-well plates (Sarstedt, Nümbrecht, Germany) in mammosphere formation medium (200 μl) supplemented with the appropriate concentration of PS particles (64,000 p/ml and 4,000 p/ml for all three PS particle granularities). The mammosphere formation medium consisted of medium I and medium II at a ratio of 1:4. Medium I was DMEM/F12 (Pan Biotech, Aidenbach, Germany) supplemented with 6.6 % B27 supplement (Thermo Fisher Scientific, Wesel, Germany), 20 ng/ml FGF (human recombinant; Merck KGaA, Darmstadt, Germany), 20 ng/ml EGF (human recombinant; Merck KGaA, Darmstadt, Germany) and 0.39 μg/ml hydrocortisone (Sigma‒Aldrich, Taufkirchen, Germany). Medium II consisted of DMEM (Pan Biotech, Aidenbach, Germany) and Methocult H4100 (Stem Cells Technologies, Cologne, Germany) at a ratio of 3:2. Mammospheres were cultured for up to 10 days in a humidified atmosphere at 37 °C with 5 % CO_2_. The mammosphere formation capacity was determined using an Incucyte® SX5 Live-Cell Analysis System (Sartorius, Göttingen, Germany), whereby mammospheres with a diameter <60 μm were excluded from analysis.

### 2.9 Scratch wound healing assay

M13SV1_Syn1-DSP8-11, HS578T-DSP1-7 and MDA-MB-231-DSP1-7 cells (2 × 10^5^ cells per well) were seeded in triplicate in 24-well plates (Sarstedt, Nümbrecht, Germany) and cultured at 37 °C with 5 % CO_2_ in a humidified atmosphere until they reached 100 % confluence (usually after 24–48 h). A 100 μl pipette tip was used to establish the scratch wound. The cells were washed once with PBS to remove cell debris. Thereafter, fresh medium (1.5 ml) supplemented with the appropriate concentration of PS particles (64,000 p/ml and 4,000 p/ml for all three PS particle granularities) was applied. The closing of the scratch wound was recorded using an Incucyte® SX5 LiveCell Imaging system (Sartorius AG, Göttingen, Germany) and analyzed using Fiji (ImageJ) software (https://imagej.net/software/fiji/downloads). Transmission light microscopy images were captured every 4 h for a total time of 24 h. The scratch wound closure rate (%) was calculated by determining the scratch wound area at t = 0, 4, 8, 12, 16, 20, and 24 h. Then, the scratch wound area at t = 4, 8, 12, 16, 20, and 24 h was calculated in relation to the scratch wound area at t = 0 h, which was set to 0 %.

### 2.10 Confocal laser scanning microscopy

Confocal laser scanning microscopy analyses were conducted to visualize the absorption of the green fluorescent-labeled PS particles, and their intracellular/cytosolic distributions (Leica TCS SP5; Leica Microsystems, Wetzlar, Germany) were determined. M13SV1_Syn1-DSP8-11, HS578T-DSP1-7 and MDA-MB-231-DSP1-7 cells (2 × 10^4^ cells per well) were seeded in chamber slides and incubated for 24 h in a humidified atmosphere at 37 °C with 5 % CO_2_. Then, the medium was replaced with medium supplemented with 64,000 PS particles/ml, and the cells were cultivated for an additional 24 h. In these experiments, only 0.5 μm and 1.0 μm green fluorescent-labeled PS particles were used. The cells were washed once with PBS, fixed with 4 % paraformaldehyde (Merck KGaA, Darmstadt, Germany) for 20 min at room temperature and washed twice with PBS. Then, the cells were permeabilized with 0.5 % Triton (v/v in PBS) for 5 min at room temperature and washed three times with PBS. Alexa Fluor® 568-phalloidin (Thermo Fisher Scientific, Wesel, Germany) was used to stain the actin cytoskeleton (30 min, room temperature). Finally, the cells were thoroughly washed with PBS and mounted with Fluoromount (Thermo Fisher Scientific, Wesel, Germany).

In addition to the microscopy images, so-called Z-stacks, i.e., a series of images through the sample, were prepared for 3D reconstructions. The optimal section thickness for the Z-stacks of the 3D reconstructions was 0.13 μm (130 nm), which was previously determined using the Nyquist Rate Calculator (https://3roam.com/nyquist-frequency-calculator/). Huygens deconvolution software (Scientific Volume Imaging B.V., Hilversum, The Netherlands) was used for image optimization and the creation of 3D models. The simulated fluorescence process mode (SFP mode) was selected for image visualization. Huygens software was also used to create videos of the 3D cell models so that an improved spatial representation of the recorded PS particles could be generated.

### 2.11 Statistics

Statistical analysis was performed using GraphPad PRISM software 9.5.0 (graphpad.com). All data are shown as the means ± standard errors of the means (S.E.M.s). p values were determined using one-way or two-way analysis of variance (ANOVA) and Tukey's post hoc test. The following p values indicated significant differences between groups: ∗p < 0.0332, ∗∗p < 0.0021, ∗∗∗p < 0.0002, and ∗∗∗∗p < 0.0001.

## 3 Results

### 3.1 Human breast epithelial cells and human breast cancer cells take up PS particles in a dose- and size-dependent manner

**Fig. 1:**
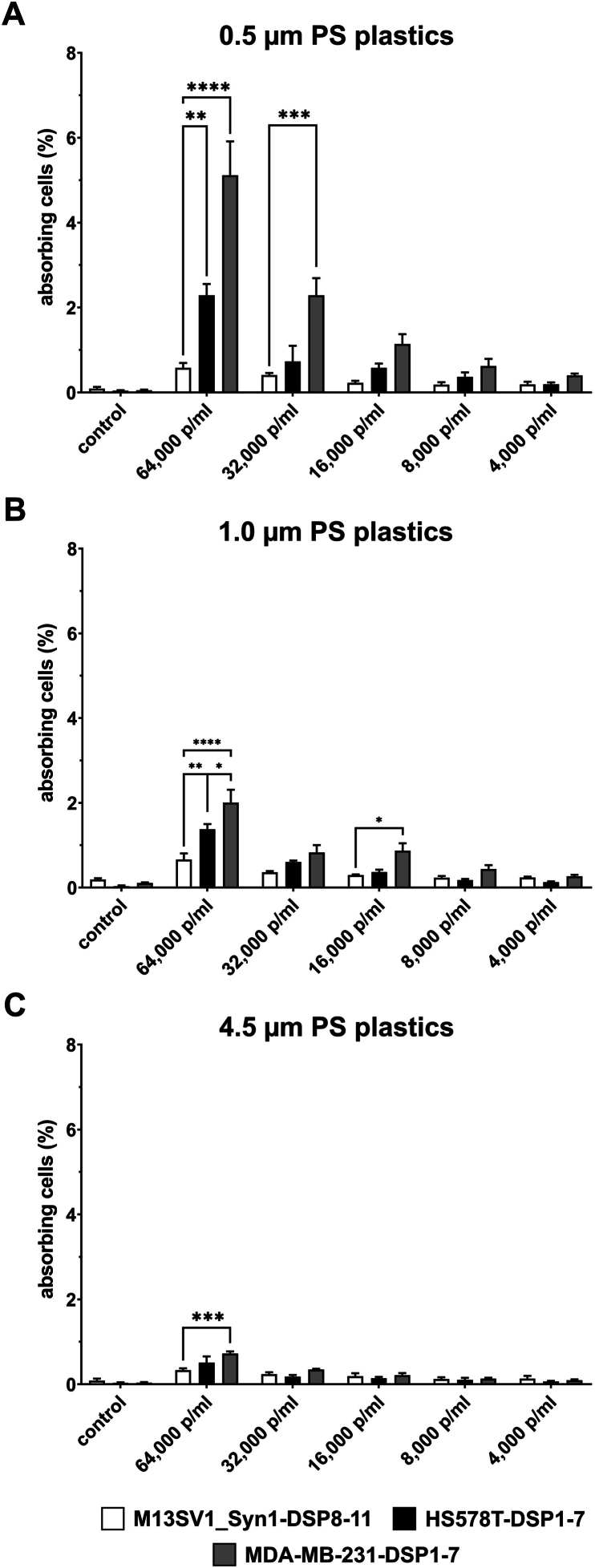
Evaluation of particle absorption by M13SV1_Syn1-DSP8-11, HS578T-DSP1-7 and MDA-MB-231-DSP1-7 cells using flow cytometry. A) PS beads with sizes of 0.5 μm, **B)** 1.0 μm, and **C)** 4.5 μm. The flow cytometry data (means ± S.E.M.s) from three independent experiments are shown (n = 3). Statistical significance was determined using two-group ANOVA and Tukey's post hoc test: ∗p < 0.0332, ∗∗p < 0.0021, ∗∗∗p < 0.0002, and ∗∗∗∗p < 0.0001.

M13SV1_Syn1-DSP8-11 human breast epithelial cells and HS578T-DSP1-7 and MDA-MB-231-DSP1-7 human breast cancer cells were cultivated with three different PS particle sizes (0.5 μm, 1.0 μm and 4.5 μm) and five different concentrations (64,000 p/ml, 32,000 p/ml, 16,000 p/ml, 8,000 p/ml and 4,000 p/ml) for 24 h. The absorption of green-fluorescing PS particles was quantified by flow cytometry. The results are summarized in Fig. 1 and clearly show the dose-, size-, and cell line-dependent absorption of the PS particles.

The higher the concentration of PS particles was, the greater the number of cells that internalized the PS particles. Similarly, smaller PS particles were better absorbed than larger particles were. This result was the same for all the cell lines examined (Fig. 1A–C). Interestingly, cell line-specific differences were observed in the absorption of PS particles. The highest absorption of PS particles was always observed in MDA-MB-231-DSP1-7 breast cancer cells. In terms of the highest PS particle concentration (64,000 p/ml), 5.7 ± 1.3 % of the MDA-MB-231-DSP1-7 cells were positive for 0.5 μm particles, 2.0 ± 0.5 % of the cells were positive for 1 μm particles, and 0.8 ± 0.1 % of the cells were positive for 4.5 μm particles (Fig. 1A–C). In contrast, M13SV1_Syn1-DSP8-11 breast epithelial cells absorbed the fewest PS particles. The highest particle concentrations (64,000 p/ml) were 0.6 ± 0.2 % (0.5 μm), 1.0 ± 0.5 % (1 μm) and 0.3 ± 0.1 % (4.5 μm). The absorption capacity of HS578T-DSP1-7 breast cancer cells was between that of MDA-MB-231-DSP1-7 and M13SV1_Syn1-DSP8-11 cells (Fig. 1A–C). Notably, for the 1 μm PS particles, a relatively constant absorption capacity of 0.6–1.0 % was observed for the M13SV1_Syn1-DSP8-11 cells at all the particle concentrations used (Fig. 1B).

### 3.2 Visualization of PS particle absorption by confocal laser scanning microscopy

**Fig. 2:**
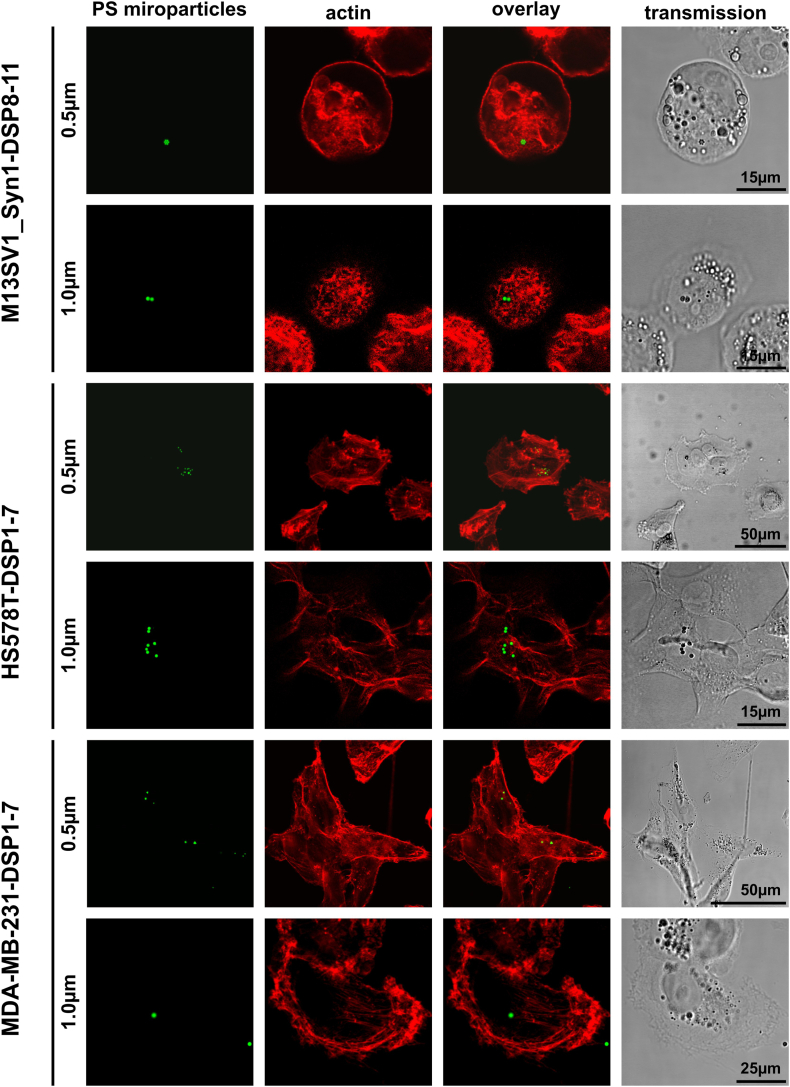
Visualization of PS particle absorption by M13SV1_Syn1-DSP8-11, HS578T-DSP1-7 and MDA-MB-231-DSP1-7 cells via confocal laser scanning microscopy. Representative data from two independent experiments are shown. Computer-based 3D reconstructions of the 1.0 μm PS particles incorporated into M13SV1_Syn1-DSP8-11, HS578T-DSP1-7 and MDA-MB-231-DSP1-7 cells are provided in the Supplementary data.

Confocal laser scanning microscopy analyses were performed to qualitatively visualize the absorption of green fluorescent PS particles by M13SV1_Syn1-DSP8-11 human breast epithelial cells and HS578T-DSP1-7 and MDA-MB-231-DSP1-7 human breast cancer cells. Due to the relatively low absorption of the 4.5 μm PS microparticles in all the cell lines, 0.5 μm and 1 μm PS microparticles at the highest concentration of 64,000 microparticles/ml were used in these experiments. The actin cytoskeleton was counterstained with phalloidin to visualize the cell boundaries and the absorption of microparticles, and for some analyzed cells, Z-stack images were taken for computer-based 3D reconstructions (Supplementary data). Briefly, the absorption of 0.5 μm and 1.0 μm PS microparticles was observed in all the cell lines (Fig. 2), which was further confirmed by computer-based 3D reconstructions (1.0 μm; Supplementary data).

### 3.3 Determination of the sizes of breast epithelial cells and breast cancer cells

**Fig. 3:**
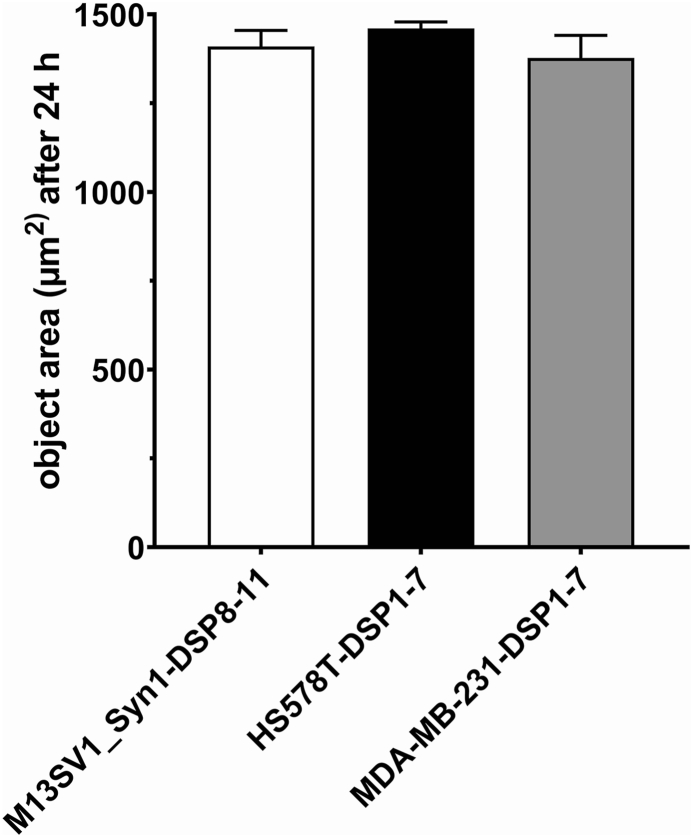
Relative sizes of M13SV1_Syn1-DSP8-11, HS578T-DSP1-7 and MDA-MB-231-DSP1-7 cells. The data are presented as the means ± S.E.M.s of three independent experiments. No significant differences were observed (one-way ANOVA and Tukey's post hoc test).

The relative size of the cells used was determined based on the proliferation data to exclude the possibility that the greater absorption of PS particles in both types of breast cancer cells than in breast epithelial cells was due to a larger size of the cancer cells. The results are summarized in Fig. 3 and clearly show comparable sizes for M13SV1_Syn1-DSP8-11, HS578T-DSP1-7 and MDA-MB-231-DSP1-7 cells. Thus, the increased absorption of PS particles by MDA-MB-231-DSP1-7 and HS578T-DSP1-7 breast cancer cells may be related to other processes.

### 3.4 Effect of PS particles on cell proliferation

**Fig. 4:**
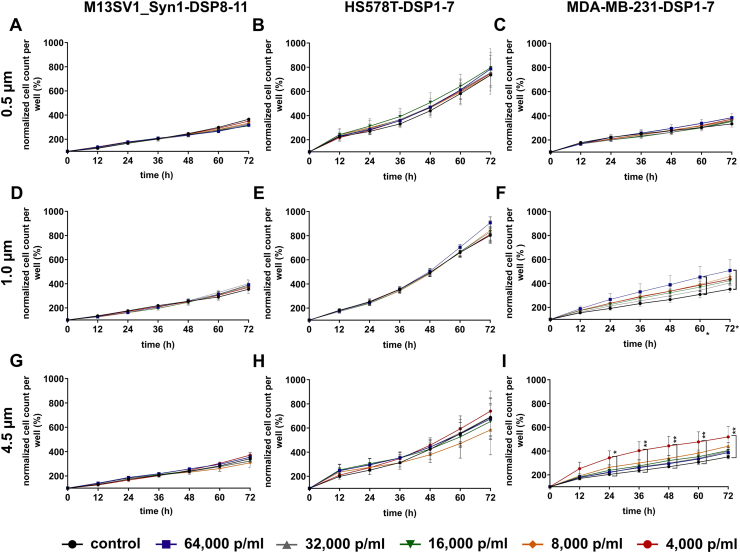
Effect of PS particles on the proliferation of M13SV1_Syn1-DSP8-11, HS578T-DSP1-7 and MDA-MB-231-DSP1-7 cells. The data are presented as the means ± S.E.M.s of three independent experiments. No significant differences were observed (two-way ANOVA and Tukey's post hoc test).

The prospective effect of PS particles on the proliferation of M13SV1_Syn1-DSP8-11 human breast epithelial cells and HS578T-DSP1-7 and MDA-MB-231-DSP1-7 human breast cancer cells was investigated using the IncuCyte® SX5 LiveCell Imaging system. The cell proliferation data are summarized in Fig. 4 and show that the cells responded differently to the PS particles. For M13SV1_Syn1-DSP8-11 human breast epithelial cells, no effect of PS particles on cell proliferation was observed (Fig. 4A–D, G).

In contrast to the findings in M13SV1_Syn1-DSP8-11 cells, a slight effect of PS particles on cell proliferation was observed in both breast cancer cell lines, although this effect was significant only in MDA-MB-231-DSP1-7 cells treated with 1.0 μm and 4.5 μm PS particles (Fig. 4F, I). For example, the proliferation of MDA-MB-231-DSP1-7 cells increased in the presence of all concentrations of the 1.0 μm PS particles (Fig. 4F). A significant increase was observed for the highest particle concentration (64,000 p/ml) after 60 and 72 h (Fig. 4F). Even after treatment with 4.5 μm PS particles, all the treated MDA-MB-231-DSP1-7 cells presented an increased proliferation rate, with the highest and significant proliferation rate observed for the MDA-MB-231-DSP1-7 cells treated with 4,000 PS p/ml (Fig. 4I). Interestingly, this effect was reproducible. The observation that only the breast cancer cell lines responded to PS particles, and not the human breast epithelial cell line, fits well with the flow cytometry data, which showed that the PS particles were internalized primarily by the breast cancer cell lines.

### 3.5 Effect of PS particles on the colony formation capacity

**Fig. 5:**
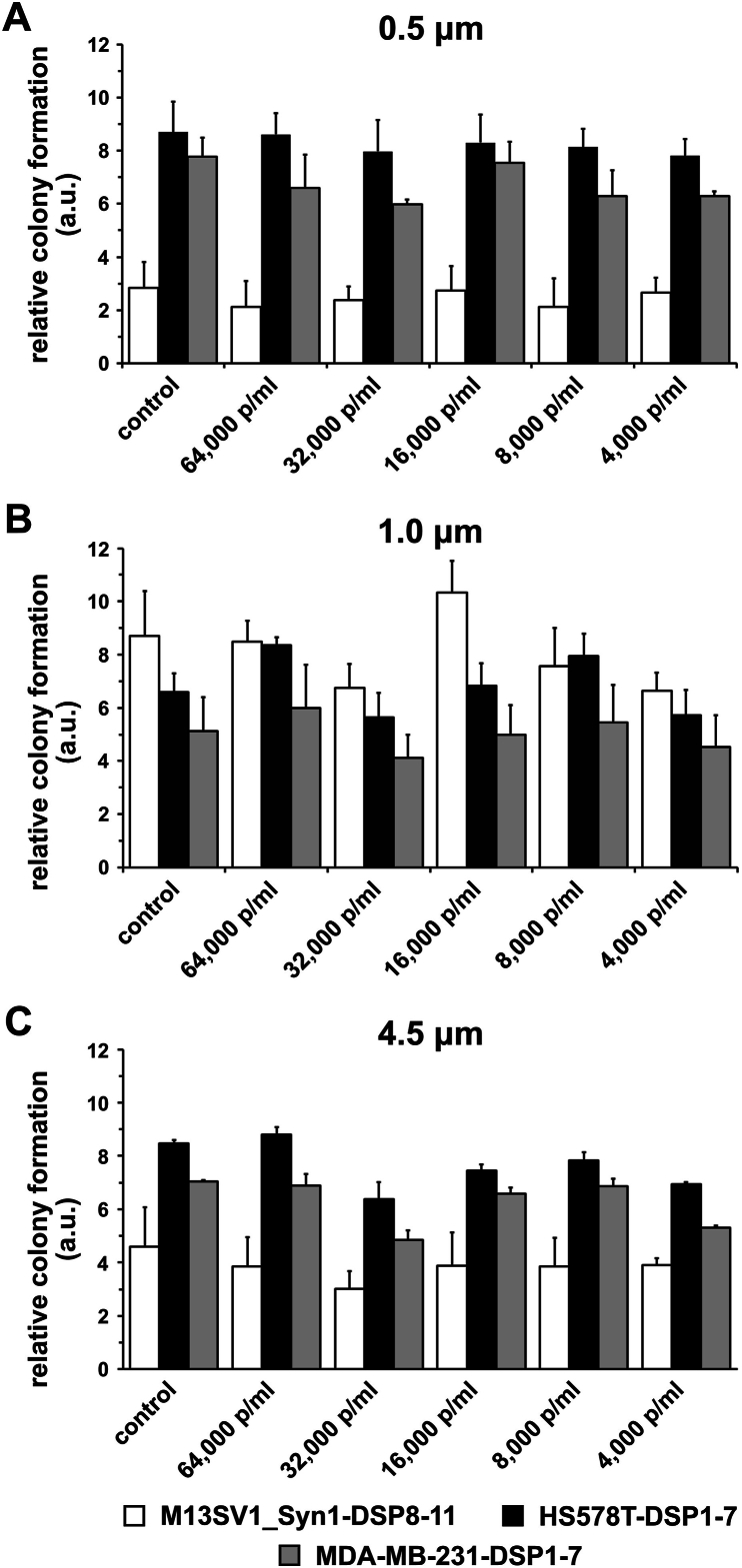
Effect of PS particles on the colony formation capacity of M13SV1_Syn1-DSP8-11, HS578T-DSP1-7 and MDA-MB-231-DSP1-7 cells. The data are presented as the means ± S.E.M.s of three independent experiments. No significant differences were observed (one-way ANOVA and Tukey's post hoc test).

The colony formation assay is an in vitro quantitative technique used to examine the ability of a single cell to grow into a large colony through clonal expansion and is thus commonly used as a sensitive indicator of the prospective stemness properties of cancer cells [42]. The cells were seeded at a low density (200 cells per well) and treated for up to 10 days with different PS particle sizes and concentrations to test whether PS particles may affect the colony formation capacity of M13SV1_Syn1-DSP8-11, HS578T-DSP1-7 and MDA-MB-231-DSP1-7 cells. The data are summarized in Fig. 5 and show that the colony formation capacity of the cells was not influenced by PS particles. The colony formation capacity of the control cells was similar to that of the PS particle-treated cells. Although differences in colony formation capacity between the different PS particle sizes were observed (e.g., M13SV1_Syn1-DSP8-11 cells formed more colonies after treatment with 1.0 μm PS particles (control: 8.7 ± 1.7; Fig. 5B) than after treatment with the 0.5 μm (control: 2.8 ± 1.0; Figs. 5A) and 4.5 μm particles (control: 4.6 ± 1.5; Fig. 5C) experiments), this difference could not ultimately be attributed to the PS particles themselves, as the corresponding differences were also observed in control cells.

### 3.6 Effect of PS particles on cell‒cell fusion

**Fig. 6:**
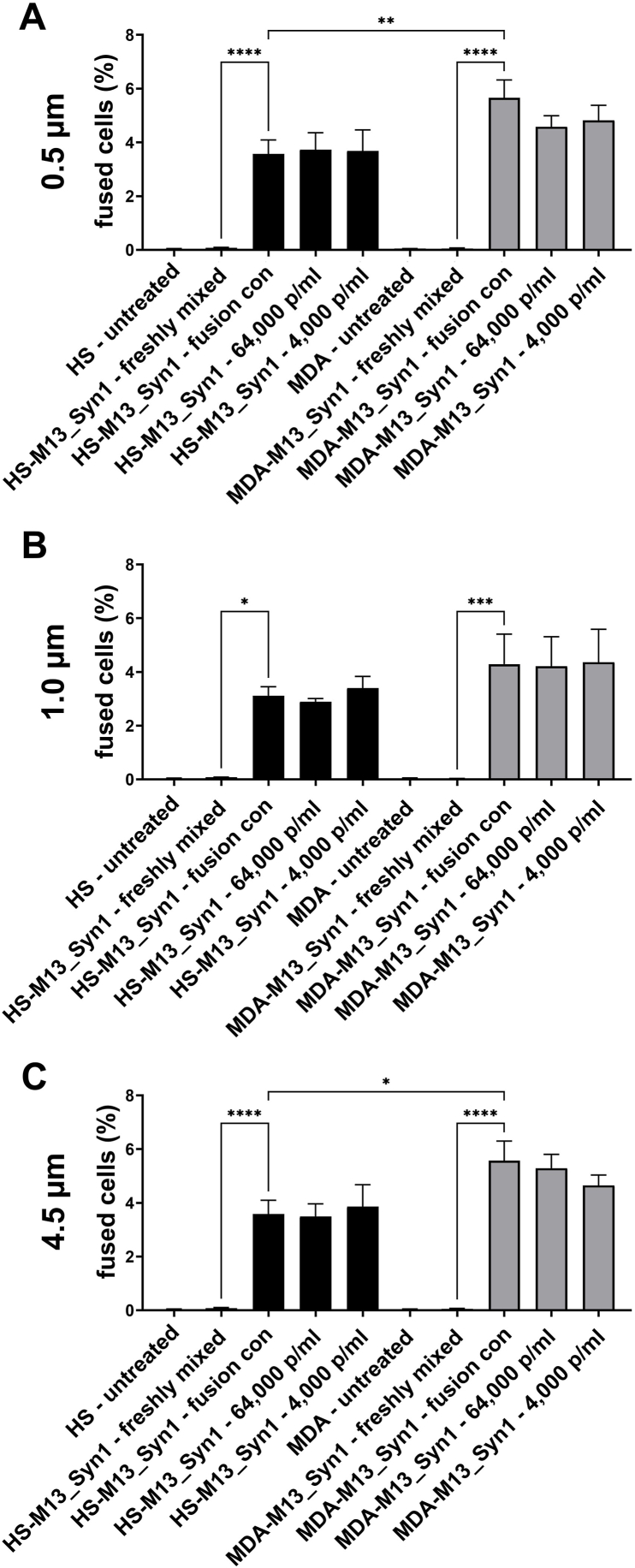
Effect of PS particles on the cell‒cell fusion of M13SV1_Syn1-DSP8-11 with HS578T-DSP1-7 cells and M13SV1_Syn1-DSP8-11 with MDA-MB-231-DSP1-7 cells. The data are presented as the means ± S.E.M.s of three independent experiments. Statistical significance was determined using two-group ANOVA and Tukey's post hoc test: ∗p < 0.0332, ∗∗p < 0.0021, ∗∗∗p < 0.0002, and ∗∗∗∗p < 0.0001.

Cell‒cell fusion has been suggested as a potential driving force for increasing intratumoral heterogeneity due to the induction of polyploidy and aneuploidy [[43], [44], [45], [46], [47]]. Thus, we next analyzed whether the ability of M13SV1_Syn1-DSP8-11 human breast epithelial cells to merge with HS578T-DSP1-7 and MDA-MB-231-DSP1-7 human breast cancer cells was influenced by PS particles. A dual split (DSP) assay was performed to quantify cell‒cell fusion events via flow cytometry [40,41]. Quantification of cell‒cell fusion revealed no effect of the PS particle size or concentration on cell fusion. However, the data revealed that the spontaneous fusion frequency of untreated MDA-MB-231-DSP1-7 and M13SV1_Syn1-DSP8-11 cells was slightly higher than the spontaneous fusion frequency of HS578T-DSP1-7 and M13SV1_Syn1-DSP8-11 cells. Treatment of MDA-MB-231-DSP1-7 and M13SV1_Syn1-DSP8-11 cells with either 4,000 or 64,000 0.5 μm p/ml or 4,000 4.5 μm microparticles, respectively, resulted in a slight but not significant decrease in the cell‒cell fusion rate (Fig. 6A–C).

### 3.7 Effect of PS particles on the mammosphere formation capacity

The mammosphere formation assay is another method for investigating the potential stem cell properties of (breast) cancer cells [48]. In addition to the mean number of formed mammospheres, the average mammosphere size represents another parameter. As the 4.5 μm PS particles were poorly absorbed by the cells (Fig. 1C), only 0.5 μm and 1.0 μm PS particles were used in the mammosphere formation assay, and the concentrations used were 64,000 and 4,000 p/ml, respectively.

**Fig. 7:**
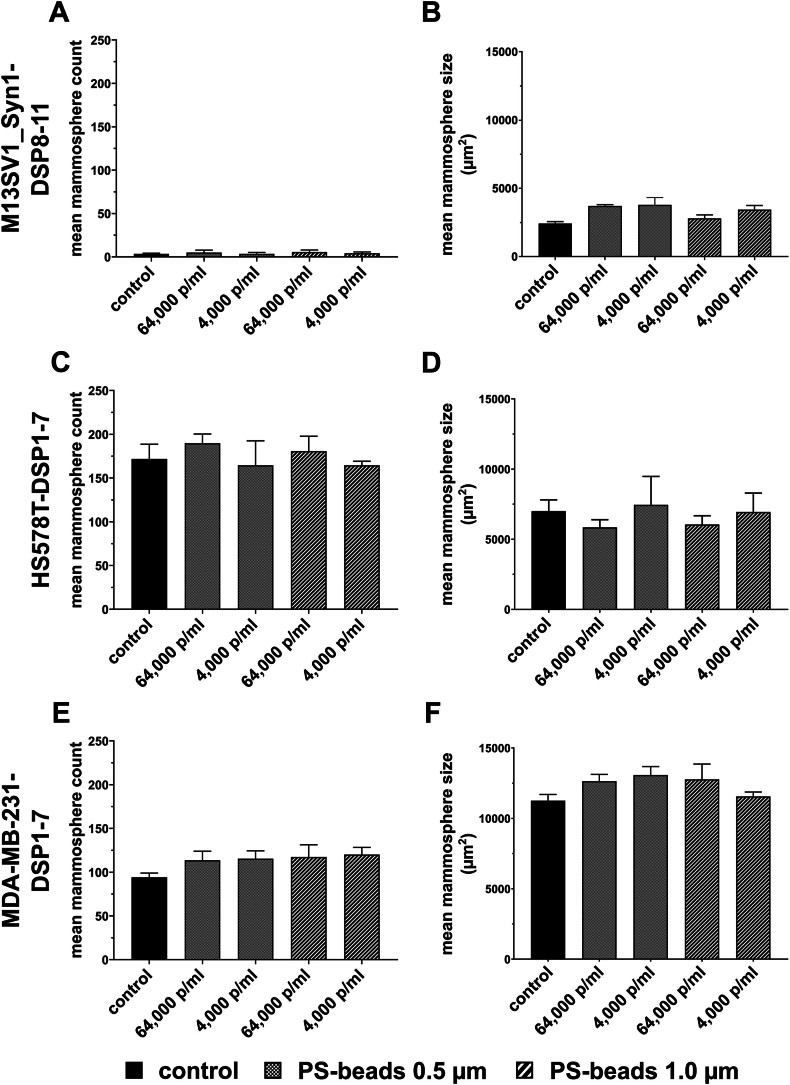
Effect of PS particles on the mammosphere formation capacity of M13SV1_Syn1-DSP8-11, HS578T-DSP1-7 and MDA-MB-231-DSP1-7 cells. The data are presented as the means ± S.E.M.s of three independent experiments. The data indicate that both the mean mammosphere count and size of MDA-MB-231-DSP1-7 breast cancer cells were slightly but not significantly increased by PS particles. No significant differences were observed (one-way ANOVA and Tukey's post hoc test).

In accordance with previous studies [49,50], only a very few rather small mammospheres were derived from M13SV1_Syn1-DSP8-11 human breast epithelial cells (Fig. 7A and B). As expected, mammospheres were derived from both HS578T-DSP1-7 and MDA-MB-231-DSP1-7 human breast cancer cells; HS578T-DSP1-7 cells produced significantly more mammospheres than did the MDA-MB-231-DSP1-7 cells (172 ± 17 vs. 94 ± 5; p < 0.05; Fig. 7C–E). Interestingly, a slightly increased mammosphere number formed from HS578T-DSP1-7 cells treated with 64,000 p/ml PS particles (0.5 μm: 190 ± 10; 1.0 μm: 181 ± 17; Fig. 6C), whereas the number of mammospheres that formed after treatment with 4,000 p/ml PS particles was comparable to the control values (0.5 μm: 164 ± 28; 1.0 μm: 165 ± 5; Fig. 7C). Conversely, the number of HS578T-DSP1-7-derived mammospheres was slightly greater in the presence of 4,000 PS particles/ml than in the presence of 64,000 PS particles/ml (Fig. 7D). In contrast, slightly increased numbers of mammospheres formed from the MDA-MB-231-DSP1-7 cells treated with both 0.5 μm and 1.0 μm PS particles at 64,000 and 4,000 p/ml (0.5 μm: 64,000 p/ml: 114 ± 10 and 4,000: 115 ± 9; 1.0 μm: 64,000: 117 ± 14 and 4,000: 120 ± 8; Fig. 7E). Similarly, slightly larger mammospheres were derived from PS particle-treated MDA-MB-231-DSP1-7 breast cancer cells (Fig. 7F). These results were reproducible and might indicate the potential effect of PS particles on the mammosphere formation capacity of human breast cancer cells.

### 3.8 Effect of PS particles on cell migration

**Fig. 8:**
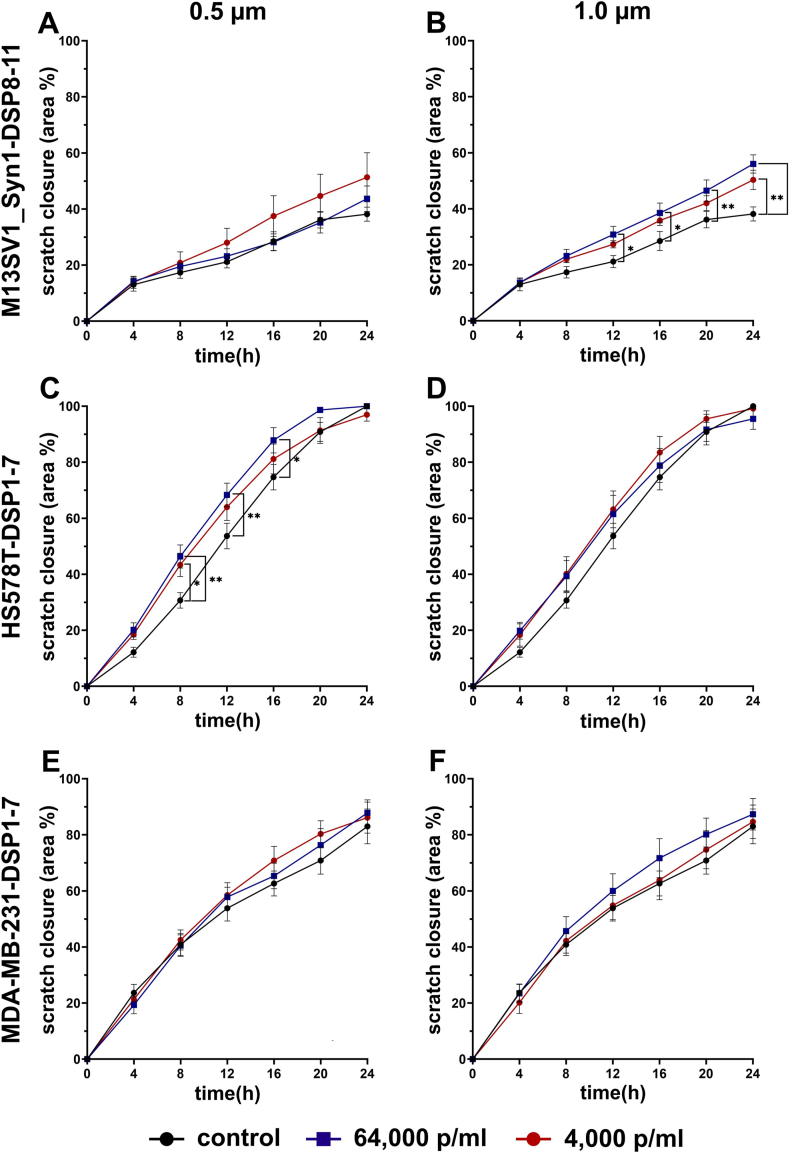
Effect of PS particles on the migration of M13SV1_Syn1-DSP8-11, HS578T-DSP1-7 and MDA-MB-231-DSP1-7 cells. The data are presented as the means ± S.E.M.s of three independent experiments. Statistical significance was determined using two-group ANOVA and Tukey's post hoc test: ∗p < 0.0332 and ∗∗p < 0.0021.

A scratch wound healing assay was performed to study the effect of PS particles on cell migration. In accordance with the results of the mammosphere formation assay, only 0.5 μm and 1.0 μm PS particles at concentrations of 64,000 and 4,000 p/ml were used because of the poor absorption of the 4.5 μm PS particles (Fig. 1C). The data are summarized in Fig. 8 and indicate that the migration of M13SV1_Syn1-DSP8-11, HS578T-DSP1-7 and MDA-MB-231-DSP1-7 cells was partially significantly increased in the presence of PS particles. For example, the scratch wounds in the confluent M13SV1_Syn1-DSP8-11 monolayers were closed by 50 ± 3 % (4,000; 1.0 μm p/ml) and 56 ± 3 % (64,000; 1.0 μm p/ml), respectively, after 24 h, which was significantly faster than that in the untreated control cells (38 ± 3 %; Fig. 8B). Similarly, significantly faster scratch wound closure was observed for HS578T-DSP1-7 cells treated with 0.5 μm and 1.0 μm PS particles (4,000 p/ml and 64,000 p/ml; Fig. 8C). In contrast to HS578T-DSP1-7 cells, only moderate impacts of 0.5 μm and 1.0 μm PS particles on the migration of MDA-MB-231-DSP1-7 cells were observed. Although the differences between the control cells and cells treated with PS particles were rather small and not significant, we always observed faster scratch wound closure in the presence of 64,000 p/ml PS particles (Fig. 8E and F).

## 4 Discussion

In the present study, we investigated the effects of PS particles on M13SV1_Syn1-DSP8-11 human breast epithelial cells, as well as HS578T-DSP1-7 and MDA-MB-231-DSP1-7 human breast cancer cells. Our data indicate that PS particles are absorbed by these cells in a dose- and size-dependent manner, which is consistent with other reports [15,16,23,24,29,30,51,52]. Interestingly, we also observed a cell line-specific effect, whereby the absorption of PS particles was much more efficient in MDA-MB-231-DSP1-7 breast cancer cells than in M13SV1_Syn1-DSP8-11 human breast epithelial cells (Fig. 1). Five routes of the cellular absorption of plastic particles are known: i) phagocytosis, ii) macropinocytosis, iii) clathrin-mediated endocytosis, iv) caveolae-mediated endocytosis and v) passive diffusion [32,53]. Studies by Voronovic and colleagues revealed that citrate-coated 54 nm × 41 nm rare-earth-doped nanoparticles were adsorbed via clathrin-mediated endocytosis in MDA-MB-231 breast cancer cells but by caveolin-mediated endocytosis in MCF-7 breast cancer cells [29]. In contrast, the absorption of silica-coated nanoparticles in both breast cancer cell lines was achieved via macropinocytosis [29]. Liu et al. and Bonanomi et al. used different specific inhibitors to block endocytosis, phagocytosis or macropinocytosis to clarify the mechanisms through which nano- and microparticles are internalized in cells [28,54]. For example, 50 nm PS nanoplastics were shown to be internalized by clathrin- and caveolin-mediated endocytosis, as well as macropinocytosis, whereas 500 nm PS nanoplastics were internalized only by macropinocytosis in RBL-2H3 rat basophilic leukemia cells [54]. Interestingly, in addition to active endocytosis, passive internalization via diffusion was also documented for 50 nm and 500 nm PS nanoparticles [54]. However, other studies have shown that the upper size limit for clathrin-mediated endocytosis of Fluoresbrite® PS nanoplastics tends to be 200 nm, whereas larger Fluoresbrite® PS nanoplastics >500 nm in size are absorbed via caveolin-mediated endocytosis in B16‒F10 murine melanoma cells [55], which contrasts with the findings of Liu and colleagues [54]. Data from Chaves et al. indicate that iron oxide citrate nanoparticles with a mean hydrodynamic diameter of 40–200 nm are internalized by clathrin-mediated endocytosis in MDA-MB-231 and MCF-7 breast cancer cells [56]. Therefore, we can speculate only on the mechanism by which the PS particles were absorbed into the cells used in this study. In this context, PS nanoparticles are most likely absorbed via clathrin- and caveolin-dependent endocytosis and PS microparticles are rather absorbed via micro- and macropinocytosis or phagocytosis [32,53,55]. Nonetheless, the absorption of PS particles by M13SV1-Syn1-DSP8-11, HS578T-DSP1-7 and MDA-MB-231-DSP1-7 cells should be clarified in future studies using specific inhibitors that target clathrin-mediated endocytosis, caveolae-mediated endocytosis and macropinocytosis.

Here, the cells were treated with different concentrations of PS particles ranging from 4,000 p/ml to 64,000 p/ml. These concentrations correspond to the aforementioned range of the estimated daily absorption of nano- and microplastics of approximately 4,594–94,500 particles [25,26]. Moreover, in the study by Visalli et al., HT29 colon carcinoma cells were treated with PS microplastic suspensions ranging from 100 to 1,600 p/ml [30]. This particle count was sufficient to observe an effect on the examined HT29 cells. Furthermore, the cell count in the work of Visalli et al. was between 1 × 10^4^ and 2 × 10^4^ [30], which is also comparable to our studies. In contrast, many other studies have used defined quantities (μg/ml) of nano- and microplastics [15,16,23,24,51,54]. For example, in the study by Liang and colleagues, mice were treated with a single dose of PS nano- or microplastics ranging from 125 to 500 mg/kg body weight in biodistribution experiments [24]. Within 24 h, 50-nm and 500-nm PS particles were detected in the kidneys, livers and hearts of treated animals [24]. The proliferation, but not the viability, of HEK293 kidney cells and HepG2 liver cells was affected by 1.0 μm PS microplastics at a concentration of 100 μg/ml [51]. As the specific weights of the 0.5 μm, 1.0 μm and 4.5 μm PS particles were not provided by the manufacturers, comparisons with studies that used defined quantities of nano- and microplastics are difficult. Nonetheless, the results of Visalli and colleagues showed that even low amounts of only 800 p/ml were able to induce reactive oxygen species (ROS) production in HT29 colon cancer cells in a time-dependent manner [30]. Similarly, highly fragmented DNA was observed in HT29 cells that were treated with 1,600 p/ml PS nano- and microplastics for 24 h [30]. These findings indicate that even low concentrations of PS nano- and microplastics could have a profound effect on cellular physiology. In accordance with these findings, our data indicated that both breast cancer cell lines were susceptible to PS nano- and microparticle treatment, such as altered proliferation and cell migration rates (Fig. 4, Fig. 8). However, no particle size-specific effects were observed. While the proliferation of MDA-MB-231-DSP1-7 breast cancer cells was significantly increased by the 1.0 μm and 4.5 μm PS particles, no effect was observed for the 0.5 μm PS particles (Fig. 4), although the 0.5 μm PS particles were more efficiently absorbed than the 1.0 μm and 4.5 μm PS beads were (Fig. 1). Whether this result was attributed to unknown specific side effects of PS particles or to different routes of PS particle internalization remains unknown.

Interestingly, the cell migration data revealed slightly but partially significantly altered migratory properties of cells in the presence of 0.5 μm and 1.0 μm PS particles. Based on the cell proliferation data, how PS particles alter the migratory behavior of cells remains to be elucidated. Halimu and colleagues revealed that the epithelial-to-mesenchymal transition (EMT) and overall cell migration were induced in A549 lung carcinoma cells by PS nano- and microplastics via the upregulation of ROS production and NADPH oxidase 4 expression [57]. These results indicate an indirect PS nano- and microplastic-mediated mechanism through the induction of ROS production. In contrast, four weeks of exposure of different human gastric cancer cell lines to PS particles resulted in increased proliferation and migratory activities in all the cell lines [58]. Moreover, prolonged exposure of NCI-N87 gastric cancer cells was further associated with EMT induction, increased tumor growth, an altered gene expression pattern and increased drug resistance to bortezomib, paclitaxel, gefitinib, lapatinib, and trastuzumab [58]. Here, too, this effect was attributed to an indirect PS particle-mediated mechanism. However, whether the observed effects were possibly also caused by the induction of ROS production is unknown, as this property was not investigated in the present study. Park et al. showed that polypropylene microplastics promoted metastatic features in MCF-7 and MDA-MB-231 human breast cancer cell lines [15]. However, this effect was attributed to increased cell cycle activity and IL-6 expression but not to increased migratory activity of the cells [15]. In any case, since slightly increased migratory activity was observed in MDA-MB-231-DSP1-7 breast cancer cells in the presence of PS particles in our work, we concluded that these cells likely respond differently to different types of plastics.

Interestingly, no obvious cytotoxic effects of PS particles on M13SV1_Syn1-DSP8-11 human breast epithelial cells or HS578T-DSP1-7 and MDA-MB-231-DSP1-7 breast cancer cells were observed in this work. Cell proliferation studies were performed using the IncuCyte® live-cell imaging system, which allows continuous observation of the morphology and proliferation of cells over a longer period of time (up to 72 h). In fact, we did not observe increased cell death in PS particle-treated cells. Given that 0.5 μm PS particles were markedly better absorbed by all investigated cell lines than 4.5 μm PS particles were, one would have assumed a prospective cytotoxic effect on this cohort. This result, however, was not observed here. In any case, our results contrast with data indicating the putative cytotoxicity of PS particles [24,30,[59], [60], [61]]. For example, Visalli and colleagues reported that PS particle concentration-dependent moderate cytotoxicity in HT29 colon cancer cells was most likely related to ROS production and DNA damage [30]. Similarly, studies of Wistar rats revealed a correlation between PS microplastics and cardiovascular toxicity due to the induction of cardiac fibrosis via the activation of the Wnt/β-catenin pathway and myocardial apoptosis triggered by oxidative stress [60]. Wu et al. reported that the overall viability of CaCo-2 colon cancer cells was not altered but that both intracellular ROS generation and mitochondrial depolarization were increased by 0.1 μm and 5 μm PS nano- and microparticles [61]. In contrast, polypropylene microplastics are not cytotoxic to MDA-MB-231 breast cancer cells [15]. However, whether these findings indicate that certain cell types might be more susceptible to the cytotoxic effects of nano- and microplastics remains unclear. Notably, Park et al. used polypropylene microplastics [15], whereas PS particles were used in this work. Similarly, no cytotoxicity assay has been conducted here, and additional studies are needed to elucidate whether, e.g., ROS production can be induced in human breast epithelial cells and human breast cancer cells by PS particles.

## 5 Conclusions

The present results revealed the granularity- and dose-dependent and cell line-specific absorption of PS particles. Although the observed effects of PS particles on the investigated cells were rather moderate, they were reproducible and indicate prospective effects of PS particles on human breast epithelial cells and breast cancer cells. The extent to which these effects are stronger at higher particle concentrations and longer incubation times is still unclear and should therefore be investigated in future studies. Such studies should also include additional PS particle-associated characteristics, such as the induction of ROS production, which has been reported for colon cancer cells [30,61], and the elucidation of the mechanism by which PS particles are absorbed. In conclusion, our data suggest that PS particles may affect human breast epithelial cells and human breast cancer cells, but further research is strongly recommended to investigate the health risks of these continuously ingested pollutants.

## Data availability

The data will be made available upon request.

## CRediT authorship contribution statement

**Maximilian Schnee:** Writing – review & editing, Investigation. **Mareike Sieler:** Writing – review & editing, Methodology. **Jessica Dörnen:** Methodology. **Thomas Dittmar:** Writing – original draft, Conceptualization.

## Declaration of competing interest

The authors declare that they have no known competing financial interests or personal relationships that could have appeared to influence the work reported in this paper.
