# Supplementary material for: Effects of polystyrene nano- and microplastics on human breast epithelial cells and human breast cancer cells
Source: Heliyon. 2024 Oct 4;10(20):e38686. doi: 10.1016/j.heliyon.2024.e38686 (PMC11497447; doi:10.1016/j.heliyon.2024.e38686)
Supplement: Multimedia component 1 [file mmc1.docx]

**Supplementary data**

Computer-based 3D reconstructions of images from Fig. 2 of the 1.0 μm PS particles incorporated into M13SV1_Syn1-DSP8-11, HS578T-DSP1-7 and MDA-MB-231-DSP1-7 cells.

Supplementary data 3D M13SV1_Syn1-DSP8-11.mp4

Supplementary data 3D HS578T-DSP1-7.mp4

Supplementary data 3D MDA-MB-231-DSP1-7.mp4
